# The use of angiotensin-converting enzyme inhibitors or angiotensin II receptor blockers may relate to the survival and walking ability in geriatric patients with hip fractures: a 1-year follow-up study

**DOI:** 10.1186/s12891-023-06362-5

**Published:** 2023-03-28

**Authors:** Qining Chu, Liqiang Wang, Qingbo Chu

**Affiliations:** Emergency Trauma Center, Nanyang Second People’s Hospital, No 66, East Jianshe Road, Nanyang473000, Nanyang, 473000 Henan China

**Keywords:** Hip fractures, ACEI, ARB, Hypertension, Prognosis

## Abstract

**Background:**

Many elder patients with hip fractures also suffered from hypertension. This study aims to explore the relationship between the use of ACEI or ARB and the outcomes of geriatric hip fractures.

**Methods:**

All the patients were divided into four groups: non-users without hypertension, non-users with hypertension, ACEI users, and ARB users. The outcomes of patients in different groups were compared. LASSO regression and univariable Cox analysis were used for variable screening. Then Cox models and Logistics models were established to identify the relationships between the use of RAAS inhibitors and outcomes.

**Results:**

ACER users (*p* = 0.016) and ARB users (*p* = 0.027) had a significantly lower survival probability than the non-users with hypertension. Non-users without hypertension, ACEI users, and ARB users may face lower 6-month and 1-year mortalities and higher 6-month and 1-year free walking rates compared with non-users with hypertension.

**Conclusion:**

Patients with the use of ACEI or ARB may face a better prognosis of hip fractures.

## Introduction

Hip fractures as one of the most severe diseases have attracted more and more attention from doctors and researchers around the world due to their high mortality and disability rate, especially in older adults [1]. Most hip fractures are always caused by a low-energy injury such as falls and slip directly while the most essential cause of hip fractures is the low bone mass density (BMD) caused by osteoporosis [2]. Facing the increasingly aging society and the high incidence of osteoporosis in older patients, hip fractures have become an intractable problem of public health [3]. Geriatric patients with hip fractures may face multiple complications, such as pneumonia, pressure sores, infections, thrombus, and so on, due to the long stay in bed even after surgery, and these complications may inhibit the recovery of motor abilities and even cause death [4]. It had been reported that many risk factors may relate to the prognosis of geriatric hip fractures, including nutrition status [5], muscle strength [6], oxidative status [7], and so on. Similarly, the use of drugs was also proved to be associated with injury risk and the outcomes of hip fracture [8, 9]. Figuring out the impact of drugs on the prognosis of hip fractures in older patients may provide us with a more advanced strategy to benefit the patients and improve their life qualities.

Angiotensin-Converting Enzyme Inhibitors (ACEI) and Angiotensin II Receptor Blockers (ARB) as two kinds of Renin–angiotensin–aldosterone system (RAAS) inhibitors were widely used for patients with hypertension [10]. Many studies had proved that RASS may play a significant role in both bone metabolism and decrease bone deterioration [11, 12], and the RAAS inhibitors may enhance bone strength and mass and decrease BMD loss through Angiotensin type 1 receptor [13], OPG/ RANKL [14], and ACE2/Ang [1–7] /Mas [15] pathways. Similarly, many population studies reported that the use of ACEI or ARB may reduce the incidence of osteoporotic fractures [16]. From a survey in China, the prevalence of hypertension in Chinese adults aged 18 years and above was 27.9%, and the number of patients with hypertension was about 244.5 million [17]. The RAAS inhibitors were one of the most commonly used hypertension drugs [18]. In clinical practice, many older patients with hip fractures may also suffer from hypertension, while to our knowledge, few studies explored the influence of ACEI or ARB on the outcome of older patients with hip fractures. In this study, the older patients who underwent surgeries due to hip fractures were enrolled and divided into four groups according to their use of RAAS inhibitors and diagnosis of hypertension, and the outcomes of patients in different groups were compared to identify the impact of ACEI or ARB on the prognosis of geriatric hip fractures.

## Methods

### Study design

This study was a retrospective cohort study conducted in Emergency Trauma Center, Nanyang Second People’s Hospital. We declare that this study, as an observational study, was conducted in accordance with the Declaration of Helsinki, and approved by the Ethics Committee of Nanyang Second People’s Hospital. All the information about patient privacy was well protected, and informed consent was obtained from all patients enrolled in this study. Patients with hip fractures (diagnosed with a femoral neck fracture, intertrochanteric fracture, and subtrochanteric fracture) in our department between January 2014 and January 2021 were extracted from our hospital's electronic medical record system. The inclusion criteria were set as below: a. aged ≥ 50 years; b. underwent hip surgeries due to hip fractures. c. with low-energy injury. The exclusion criteria were: a. with pathological fractures; b. without available data about ACEI or ARB; c. with severe kidney diseases. All the patients were divided into four groups according to their use of RAAS inhibitors and hypertension: non-users without hypertension, non-users with hypertension, ACEI users, and ARB users. All surgeries were conducted by a senior doctor or in his presence and direction. All the patients were followed up for 1 year and the outcomes of patients in four groups were compared. To reduce the bias caused by co-variables, LASSO analysis was used to screen the variables and the multi-variable models including Cox models and Logistics models were established.

### Characteristics of participants and follow-up

Baseline features of patients enrolled in our study were collected and summarized, including age, sex,body mass index (BMI), type of fracture, fracture history, smoking history, alcoholism history, polytrauma, surgical procedures, anesthesia, time from injury to surgery, and so on. The fracture types were summarized as femoral neck fracture and intertrochanteric fracture, and the surgical procedures were divided into internal fixation and arthroplasty. All the total arthroplasty, bipolar and unipolar hemiarthroplasty were defined as arthroplasty. All the patients with polytrauma were not underwent surgeries for secondary injuries. The hospital examination including electrocardiogram, chest radiograph, red blood count (RBC), hemoglobin (Hb), blood glucose (GLU), and albumin (ALB) was also collected. Major comorbidities were summarized to calculate the Charlson comorbidity index (CCI), which may evaluate the pre-operative status of patients conveniently [19]. The diagnosis of hypertension was identified by the record of our electronic medical record system. The medication use of the patients after surgery was obtained by follow-up, and only the patients who used the ACEI or ARB regularly were considered users.

All of the individuals enrolled in our study were followed up for 1 year by telephone. Death was defined as all-cause death, and free walking was identified if the individuals were able to carry out activities of daily living independently. The patients who survived > 1 year were defined as censored data. The primary outcomes were mortality rates at 3 months, 6 months, and 1 year after surgery, and the second outcomes were the free walking abilities at 3 months, 6 months, and 1 year.

### Statistical analysis

Continuous variables were expressed as mean ± standard deviation and categorical variables were presented as count (percent). CCI score was transformed into a binary variable according to the cut-off value of four. Normally distributed variables were analyzed by the ANOVA test, while non-normally distributed variables were evaluated by Kruskal–Wallis test. Categorical data were assessed using Chi-squared tests.

Least Absolute Shrinkage and Selection Operator (LASSO) models were established to identify and screen the possible variables related to the 1-year mortality and 1-year free walking rate of geriatric hip fractures by using R packages “glmnet”. Firstly, the relationships between the coefficients of punishment (λ) and the weight coefficient were calculated and summarized to explore the most suitable λ. Then the λ with the strongest stability was selected by calculating partial likelihood deviance for 1-year mortality and by using the AUC method for 1-year free walking rate. Then the variables included in the LASSO models were summarized and then included in the Cox and Logistics models following.

Kaplan–Meier curves were established and the Log-rank test was used to compare the survival between groups with Bonferroni correction. Then the univariate Cox models were established and those variables that were significant were included in Cox model 1, while variables screened by LASSO models were included in Cox model 2. Similarly, the variables screened by LASSO models were also included in Logistics models to reduce the impact caused by co-variables. Due to the multicollinearity between hypertension and groups, the hypertension variable was excluded in the multi-variable models. All analyses were conducted by using R software version 4.2.2 (R Foundation for Statistical Computing, Vienna, Austria).

## Results

### Patient characteristics and outcomes

**Fig. 1:**
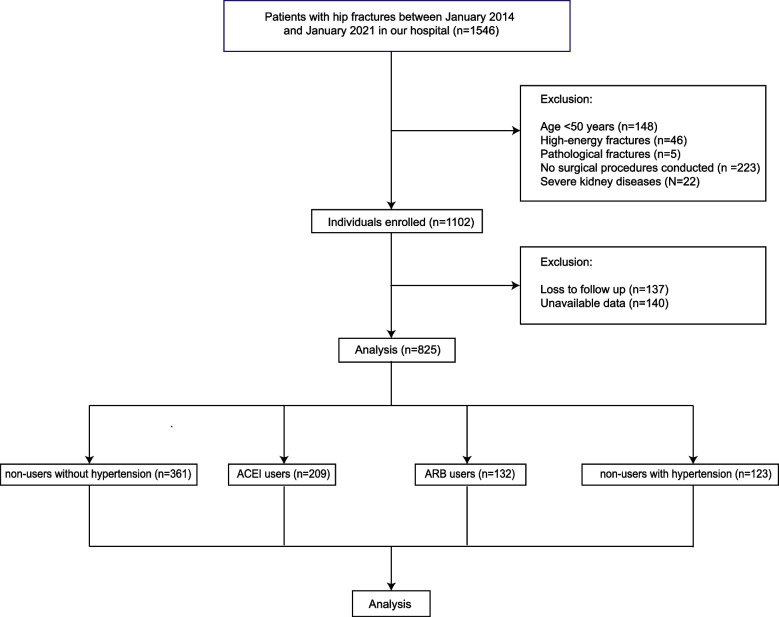
Flow chart of our study

**Fig. 2:**
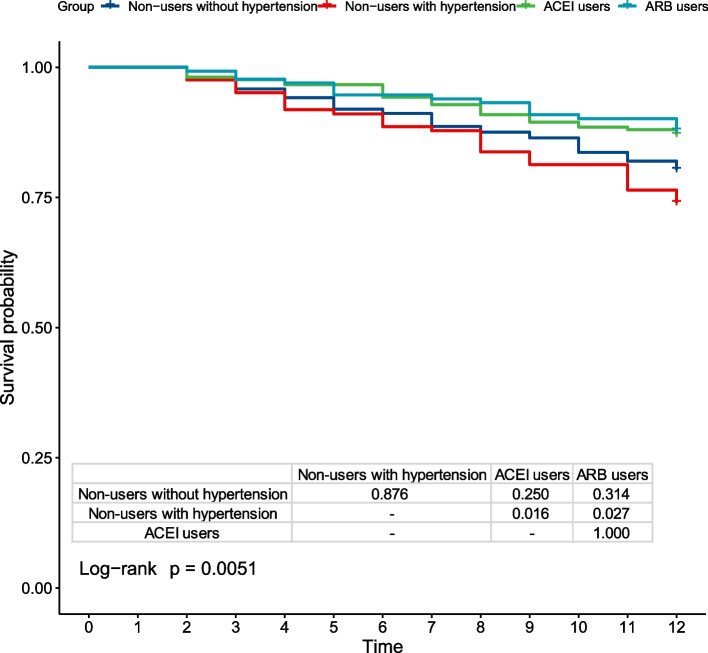
Kaplan–Meier analysis of one-year survival patients grouped by the status of RAAS inhibitors using and hypertension

Finally, 825 patients who underwent hip surgeries due to hip fractures between January 2014 and January 2021 in our hospital met the inclusion and exclusion criteria and were enrolled in our study (Fig. 1). All the patients were divided into four groups according to their use of ACEI and ARB, and their hypertension status: on-users without hypertension (*n* = 361), non-users with hypertension (*n* = 123), ACEI users (*n* = 209), ARB users (*n* = 132). Then the baseline features and outcomes were compared between groups and summarized in Table 1. There are significant differences in age (*p* < 0.001), fracture history (*p* = 0.015), hypertension (*p* < 0.001), and polytrauma (*p* = 0.045) in the comparison of baseline data between groups. In the part of outcomes, patients in different groups had significantly different 1-year mortality (*p* = 0.005), 6-month independent walking rate (*p* < 0.001), and 1-year independent walking rate (*p* < 0.001). The Kaplan–Meier curves and the Log-rank test showed that the patients in different groups had significantly different survival probabilities (*p* = 0.005, Fig. 2). To explore the differences between the two groups, a Log-rank test with Bonferroni correction was performed, and as shown in Fig. 2, the non-users with hypertension had a significantly lower survival probability than ACER users (*p* = 0.016) and ARB users (*p* = 0.027), while there was no significant difference in non-users without hypertension compared with non-users with hypertension (*p* = 0.876), ACER users (*p* = 0.250) and ARB users (*p* = 0.314), as well as ACEI compared with ARB users (*p* > 0.999).

**Table 1:** Baseline characteristics of patients grouped by the status of RAAS inhibitors use and hypertension

Variables	non-users without hypertension	non-users with hypertension	ACEI users	ARB users	*p* value
	(*n* = 361)	(*n* = 123)	(*n* = 209)	(*n* = 132)	
Demographic characteristics					
Sex(female)	231 (64.0%)	83 (67.5%)	146 (69.9%)	94 (71.2%)	0.344
Age(years)	73.11 ± 10.38	66.37 ± 11.96	76.86 ± 6.98	71.58 ± 10.59	< 0.001
BMI (kg/m2)	21.84 ± 4.63	21.51 ± 3.80	21.79 ± 3.87	21.72 ± 3.71	0.938
Fractures history (yes)	57 (15.8%)	10 (8.1%)	27 (12.9%)	29 (22.0%)	0.015
Smoking history (yes)	36 (10.0%)	15 (12.2%)	23 (11.0%)	12 (9.1%)	0.845
Alcoholism history(yes)	21 (5.8%)	5 (4.1%)	12 (5.7%)	6 (4.5%)	0.852
Surgery-related variables					
Location of fracture(femoral neck)	189 (52.4%)	63 (51.2%)	111 (53.1%)	60 (45.5%)	0.524
Surgical procedures(arthroplasty)	169 (46.8%)	57 (46.3%)	103 (49.3%)	54 (40.9%)	0.508
Anesthesia (spinal)	5 (1.4%)	1 (0.8%)	0 (0.0%)	0 (0.0%)	0.197
Time from injury to surgery (Days)	4.81 ± 0.95	4.83 ± 1.03	4.97 ± 0.93	4.82 ± 0.84	0.362
CCI score (> 4)	99 (27.4%)	20 (16.3%)	51 (24.4%)	39 (29.5%)	0.057
Electrocardiogram (abnormal)	226 (62.6%)	68 (55.3%)	127 (60.8%)	68 (51.5%)	0.114
Chest radiograph (abnormal)	175 (48.5%)	61 (49.6%)	101 (48.3%)	71 (53.8%)	0.744
Hypertension(yes)	0 (0.0%)	123 (100.0%)	209 (100.0%)	132 (100.0%)	< 0.001
Polytrauma(yes)	47 (13.0%)	15 (12.2%)	44 (21.1%)	18 (13.6%)	0.045
Laboratory findings					
RBC	4.48 ± 0.48	4.38 ± 0.51	4.41 ± 0.48	4.46 ± 0.50	0.188
Hb (g/L)	95.87 ± 13.58	94.93 ± 13.32	94.09 ± 14.40	95.91 ± 15.23	0.471
ALB (g/L)	38.39 ± 8.83	37.81 ± 9.41	37.92 ± 9.22	38.34 ± 9.04	0.838
GLU (mmol/L)	6.15 ± 1.38	6.39 ± 1.41	6.25 ± 1.35	6.04 ± 1.42	0.164
Outcomes					
3-month mortality	15 (4.2%)	6 (4.9%)	5 (2.4%)	3 (2.3%)	0.478
6-month mortality	32 (8.9%)	14 (11.4%)	12 (5.7%)	7 (5.3%)	0.166
1-year mortality	71 (19.7%)	32 (26.0%)	27 (12.9%)	16 (12.1%)	0.005
3-month independent walking rate	84 (23.3%)	28 (22.8%)	47 (22.5%)	31 (23.5%)	0.996
6-month independent walking rate	168 (46.5%)	47 (38.2%)	146 (69.9%)	94 (71.2%)	< 0.001
1-year independent walking rate	252 (69.8%)	64 (52.0%)	177 (84.7%)	112 (84.8%)	< 0.001

Continuous variables were expressed as mean ± standard deviation and categorical variables were presented as count (percent). *BMI* Body mass index, *Hb* Hemoglobin, *RBC* Red blood count, *GLU* Blood glucose, *ALB* Albumin

### Variable screening

**Fig. 3:**
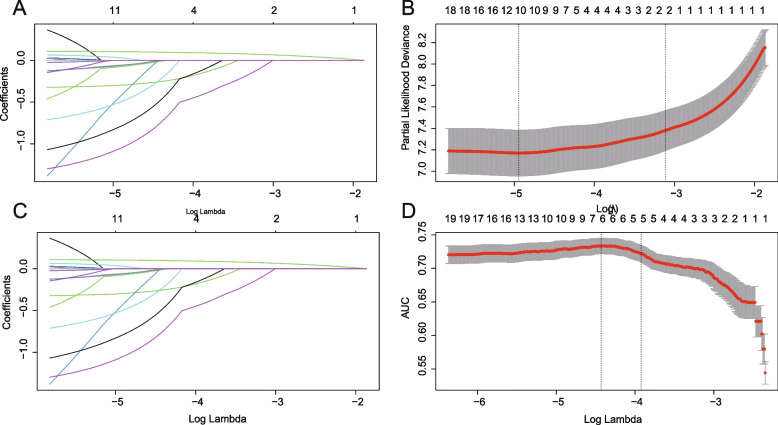
LASSO analysis for 1-year mortality and 1-year free walking rate. **A** The changes of coefficients with the increase of λ in LASSO models of 1-year mortality; **B** The changes of partial likelihood deviance with the increase of λ in LASSO models of 1-year mortality; **C** The changes of coefficients with the increase of λ in LASSO models of 1-year free walking rate; **D** The changes of AUC with the increase of λ in LASSO models of 1-year free walking rate

The direct comparison between groups may ignore the influence caused by co-variables such as ages and the bias may affect the conclusion. To identify the relationships between the use of RAAS inhibitors and the prognosis of hip fractures and to reveal the potential co-factors, LASSO models were established for 1-year mortality and 1-year free walking rate. The changes of coefficients and partial likelihood deviance with the increase of λ in LASSO models of 1-year mortality were shown in Fig. 3A and B, respectively. Similarly, the changes of coefficients and AUC were shown in Fig. 3C and D for LASSO models of 1-year free walking rate. Then the LASSO models with the minimum of λ (for 1-year mortality models: λ = 0.00712; for 1-year free walking rate models: λ = 0.01190;) were used to identify the significant variables due to the models’ highest stability. The variables included in the models were listed and summarized in Table 2. Moreover, the univariate Cox regression was also performed to screen the variables (Table 3).

**Table 2:** Variables screened by LASSO analysis

Variable	1-year mortality	1-year independent walking
	Coefficients	Coefficients
Sex(female)	-0.066	-
Age(years)	0.103	-0.011
Electrocardiogram (abnormal)	-0.300	-
Chest radiograph (abnormal)	-0.061	-
Smoking history (yes)	0.032	-
Surgical procedures(arthroplasty)	-0.079	0.051
Anesthesia (spinal)	-0.461	-
non-users with hypertension (Ref)	-	-
non-users without hypertension	-0.512	0.133
ACEI users	-1.068	0.314
ARB users	-0.807	0.256
ALB (g/L)	-	-0.001

*ALB* Albumin

**Table 3:** Cox analysis of 1-year mortality

Variables	Univariate Models		Model 1		Model 2	
	HR (95% CI for HR)	*p* value	HR (95% CI for HR)	*p* value	HR (95% CI for HR)	*p* value
Demographic characteristics						
Sex(female)	0.873 (0.622—1.225)	0.433	-	-	0.848 (0.599—1.200)	0.352
Age(years)	1.113 (1.090—1.136)	< 0.001	1.116 (1.095—1.138)	< 0.001	1.118 (1.097—1.140)	< 0.001
BMI (kg/m2)	1.003 (0.965—1.043)	0.875	-	-	-	-
Fractures history (yes)	1.085 (0.695—1.693)	0.72	-	-	-	-
Smoking history (yes)	1.190 (0.715—1.967)	0.508	-	-	1.126 (0.672—1.887)	0.652
Alcoholism history(yes)	1.036 (0.508—2.114)	0.922	-	-	-	-
Surgery-related variables						
Location of fracture(femoral neck)	1.062 (0.768—1.470)	0.715	-	-	-	-
Surgical procedures(arthroplasty)	0.937 (0.676—1.298)	0.694	-	-	0.832 (0.595—1.163)	0.281
Anesthesia (spinal)	0.000 (0.000—Inf)	0.993	-	-	0.000 (0.000—Inf)	0.994
Time from injury to surgery (Days)	1.063 (0.893—1.266)	0.494	-	-	-	-
CCI score (> 4)	1.097 (0.760—1.583)	0.62	-	-	-	-
Electrocardiogram (abnormal)	0.775 (0.559—1.074)	0.125	-	-	0.693 (0.498—0.964)	0.03
Chest radiograph (abnormal)	0.879 (0.635—1.217)	0.437	-	-	0.875 (0.628—1.218)	0.428
Polytrauma(yes)	1.171 (0.761—1.801)	0.472	-	-	-	-
Laboratory findings						
RBC (10^12/L)	1.163 (0.834—1.622)	0.373	-	-	-	-
Hb (g/L)	1.003 (0.991—1.014)	0.653	-	-	-	-
ALB (g/L)	0.994 (0.976—1.012)	0.486	-	-	-	-
GLU (mmol/L)	0.964 (0.856—1.085)	0.546	-	-	-	-
non-users with hypertension (Ref)	-	-	-	-	-	-
non-users without hypertension	0.734 (0.484—1.115)	0.147	0.405 (0.266—0.617)	< 0.001	0.426 (0.279—0.650)	< 0.001
ACEI users	0.465 (0.278—0.776)	0.003	0.220 (0.131—0.368)	< 0.001	0.230 (0.137—0.385)	< 0.001
ARB users	0.432 (0.237—0.787)	0.006	0.284 (0.156—0.518)	< 0.001	0.294 (0.161—0.539)	< 0.001

*BMI* Body mass index, *Hb* Hemoglobin, *RBC* Red blood count, *GLU* Blood glucose, *ALB* Albumin, *HR* Hazard ratio, *CI* Confidence interval. Cox model 1 was adjusted for age and groups, and Cox model 2 was adjusted for age, groups, sex, electrocardiograph, chest radiograph, smoking history, surgical procedures, and anesthesia

### Risk factors

Based on the screened variables, two Cox models were established to identify the relationship between RASS inhibitors and the prognosis of geriatric patients with hip fractures. Cox model 1 was adjusted for age and groups, and Cox model 2 was adjusted for age, groups, sex, electrocardiograph, chest radiograph, smoking history, surgical procedures, and anesthesia. As shown in Table 3, in both Cox model 1 and Cox model 2, the status of RASS inhibitors using and hypertension was the significant factor of 1-year mortalities. In univariate Cox models, compared to non-users with hypertension, ACEI users (HR = 0.456, CI: 0.278—0.776) and ARB users (HR = 0.432, CI: 0.237—0.787) had a lower risk of death, while the non-users without hypertension did not reach significance (HR = 0.734, CI: 0.484—1.115). In Cox model 1 and model 2, the association with ACEI users (Model 1: HR = 0.220, CI: 0.131 – 0.368; Model 2: HR = 0.230, CI: 0.137 – 0.385) and ARB users was sustained (Model 1: HR = 0.284, CI: 0.156 – 0.518; Model 2: HR = 0.294, CI: 0.161 – 0.539), and the non-users without hypertension was also a protective factor in adjusted models (Model 1: HR = 0.405, CI: 0.266 – 0.617; Model 2: HR = 0.426, CI: 0.279 – 0.650).

### Prognostic value

To reduce the bias caused by co-factors and identify the prognostic value of RAAS inhibitors, Logistics models for mortality and free walking rates at 3 months, 6 months, and 1 year were established and adjusted for variables screened in LASSO models (Table 4). As shown in Table 4, non-users without hypertension, ACEI users, and ARB users may face lower 6-month and 1-year mortalities and higher 6-month and 1-year free walking rates compared with non-users with hypertension. Moreover, Logistics analysis showed that ACEI users may have lower 3-month mortality compared with non-users with hypertension.

**Table 4:** Logistics analysis of mortalities and free walking abilities at 3 months, 6 months, and 1 year

Variables	3-month mortality		6-month mortality		1-year mortality	
	OR (95% CI)	*p* value	OR (95% CI)	*p* value	OR (95% CI)	*p* value
non-users with hypertension (Ref)						
non-users without hypertension	0.575 (0.212—1.748)	0.297	0.470 (0.226—1.006)	0.046	0.259 (0.136—0.486)	< 0.001
ACEI users	0.260 (0.069—0.937)	0.038	0.235 (0.096—0.563)	0.001	0.112 (0.053—0.227)	< 0.001
ARB users	0.317 (0.062—1.333)	0.13	0.292 (0.101—0.779)	0.017	0.154 (0.067—0.338)	< 0.001
Variables	3-month free walking ability		6-month free walking ability		1-year free walking ability	
	OR (95% CI)	*p* value	OR (95% CI)	*p *value	OR (95% CI)	*p* value
non-users with hypertension (Ref)						
non-users without hypertension	1.072 (0.655—1.793)	0.785	1.853 (1.192—2.912)	0.007	4.212 (2.577—6.987)	< 0.001
ACEI users	1.041 (0.597—1.841)	0.888	5.808 (3.493—9.807)	< 0.001	13.205 (7.287—24.587)	< 0.001
ARB users	1.102 (0.610—1.999)	0.748	5.393 (3.133—9.456)	< 0.001	10.156 (5.327—20.121)	< 0.001

*OR* Odds ratio, *CI* Confidence interval

## Discussion

In this study, the relationships between the use of ACEI and ARB were identified: the use of RAAS inhibitors in geriatric patients who underwent hip surgeries for hip fractures may relate to a better prognosis in both mobility and survival. To reduce the impact of co-variables and to keep the stability of Cox and Logistics models, a procedure of variable screening was conducted by using LASSO analysis. Just as the results of multi-variable regression, besides the use of ACEI and ARB, many variables were associated with the outcomes, such as age an electrocardiogram, and even after the adjustment of multi-variable models, the non-users without hypertension, ACEI users, and ARB users may also have significantly more satisfactory outcomes, which indicated that the status of RASS using and hypertension may be the independent risk factors for the prognosis of geriatric hip fractures.

Age and sex were two of the most significant risk factors for the incidence of hip fractures: the risk of hip fractures increased with age, and women may have a higher incidence of hip fractures compared with men [20, 21]. Undoubtedly, age and sex may also affect the BMD and the prognosis, so in our study, age and sex were included in the multivariable models to reduce the bias caused by them. The all-cause mortality of hip fracture in China was severe: the 1-year mortality of males was 13.69%, and the number of females was 14.77% [22]. In a study with a mean follow-up time of 38.9 months, the mortality of patients was 33.80% [23]. The cohorts in the USA showed similar results: a cohort at a level I trauma center with a two years follow-up had a 1-year mortality of 17.4% and a 2-year mortality of 24.0% [24]. Another cohort study conducted at two trauma level I centers and three community hospitals showed that 9.1% of patients died within 90 days and 23.5% within 2 years [25].

The primary outcomes of our study were all-cause death. As we know, the hip fracture itself rarely causes death directly, and usually, the development of complications of hip fractures and aggravation of comorbidity may be the major cause of death in older patients with hip fractures [26]. Hypertension as one of the most common chronic diseases was also proved to be a significant risk factor for geriatric hip fractures, and consistent with previous studies, in our study, the non-users without hypertension have a significantly lower death risk and unable free walking abilities than non-users with hypertension [27, 28]. There might be two major reasons why geriatric patients with hip fractures and hypertension may have high mortality. Firstly, hypertension itself is the most significant risk factor for death from cardiovascular diseases [29]. Moreover, hypertension may affect the survival of hip fractures through its role in bone regeneration and bone metabolism. An animal-based study showed that mice with salt-sensitive hypertension may face the reduction of femur trabecular number and bone volume fraction, and the increased number of osteoclasts and expression of RANK/OPG mRNA [30]. A meta-analysis also suggested that hypertension may relate to the reduction of BMD [31].

The relation and pathway between hypertension and bone metabolism may be achieved via the RAAS [30, 32], and the expression of RAAS components including angiotensin-converting enzyme 2 (ACE2) and Mas receptor in bone tissue, especially in osteoblasts and osteoclasts, was the foundation of the signaling pathway [33]. There also were studies that proved the expression of ACE, angiotensin type 1.receptor (AT1R), and angiotensin type 2 receptor (AT2R) in bone-healing tissue [34]. The excessive activity of the RAAS receptors in bone tissue may lead to bone resorption and inhibit bone formation [35]. Moreover, the RAAS may also affect bone regeneration with other signal factors. RAAS may increase the excretion of calcium and magnesium by interacting with parathyroid hormone (PTH) and then decrease the contents of serum calcium, which may lead to declined bone density and strength [36]. Some population studies also reported a negative relation between RAAS and vitamin D in patients with hypertension [37].

The effects of RAAS inhibitors were also reported in many studies. The mice with bilateral orchiectomy may have a significantly increased trabecular bone area after the treatment of captopril [38]. Similarly, another animal study also proved that imidapril treatment may reduce the decrease of bone density and inhibit the increase in osteoclast activation in rats with ovariectomy [39]. The effect of ACEI on bone metabolism may be achieved via the kinin-kallikrein system: the ACEI may regulate the activity levels of bradykinin [11]. The bradykinin can upregulate the expression of cyclooxygenase 2 and the biosynthesis of cytokine-induced prostaglandin and then contribute to the increase of RANKL and the activity of osteoclasts [40, 41]. In a population study, the patients with ACEI using may have a better BMD than untreated controls in a cohort of African-American elderly men [42]. Moreover, ARB also showed similar results in bone metabolism. It had been reported that ARB may significantly eliminate the levels of osteoclasts differentiation induced by Ang II and improve bone strength and microstructure in animal studies [43, 44]. In population studies, the use of ARB may significantly reduce fracture risk [16]. Recently, a meta-analysis that enrolled more than 360 million individuals indicated that the use of ACEI and ARB may both face a lower risk for fractures compared to nonusers [45]. Consistent with our study, a cohort study conducted in Scotland showed that the use of angiotensin-blocking drugs may associate with a lower risk of hip fractures and death [46].

Our study had several limitations. First, this study as a single-center retrospective study based on a small number of samples may cause bias. Next, the loss of data and follow-up may also reduce the clinical evidence grade. Thirdly, some factors that may affect the prognosis were not included in this study, and the missing co-factors may also affect the outcomes of our patients enrolled in this study. However, the co-variables which may relate to the outcomes significantly, such as ages and sex, had been included in our multi-variable models, and we believed that this may reduce the bias caused by co-variables. Lastly, the dose of RAAS inhibitors was not collected in our study, which may cause some differences in outcomes.

Our study showed that the uses of ACEI and ARB may relate to higher survival and free-walking abilities. Consistent with our study, many studies based on animals had reported the effect of RAAS inhibitors on bone healing and metabolism. We hope more experimental population studies may be conducted to prove our conclusion with a high level of evidence.

## Conclusion

Patients with the use of ACEI or ARB may face a better prognosis of hip fractures.

## Data Availability

The datasets used and/or analyzed during the current study are available from the corresponding author on reasonable request.
